# Selection and dissemination of antimicrobial resistance in Agri-food production

**DOI:** 10.1186/s13756-019-0623-2

**Published:** 2019-10-21

**Authors:** Guyue Cheng, Jianan Ning, Saeed Ahmed, Junhong Huang, Rizwan Ullah, Boyu An, Haihong Hao, Menghong Dai, Lingli Huang, Xu Wang, Zonghui Yuan

**Affiliations:** 1 MOA Laboratory for Risk Assessment of Quality and Safety of Livestock and Poultry Products, Huazhong Agricultural University, Wuhan, 430070 China; 2 National Reference Laboratory of Veterinary Drug Residues (HZAU) and MOA Key Laboratory for the Detection of Veterinary Drug Residues in Foods, Huazhong Agricultural University, Wuhan, 430070 China; 3 State key laboratory of Agricultural Microbiology, College of Veterinary Medicine, Huazhong Agricultural University, Wuhan, 430070 China

**Keywords:** Antimicrobial resistance, Co-selection, Heavy metal, Biocide, Dissemination, Antimicrobial resistance gene

## Abstract

Public unrest about the use of antimicrobial agents in farming practice is the leading cause of increasing and the emergences of Multi-drug Resistant Bacteria that have placed pressure on the agri-food industry to act. The usage of antimicrobials in food and agriculture have direct or indirect effects on the development of Antimicrobial resistance (AMR) by bacteria associated with animals and plants which may enter the food chain through consumption of meat, fish, vegetables or some other food sources. In addition to antimicrobials, recent reports have shown that AMR is associated with tolerance to heavy metals existing naturally or used in agri-food production. Besides, biocides including disinfectants, antiseptics and preservatives which are widely used in farms and slaughter houses may also contribute in the development of AMR. Though the direct transmission of AMR from food-animals and related environment to human is still vague and debatable, the risk should not be neglected. Therefore, combined global efforts are necessary for the proper use of antimicrobials, heavy metals and biocides in agri-food production to control the development of AMR. These collective measures will preserve the effectiveness of existing antimicrobials for future generations.

## Introduction

Antimicrobials, including antibiotics and related semi synthetic or synthetic drugs exhibit high antimicrobial potency and selective toxicity to allow their use as anti-infective agents [1]. Over the years, antimicrobials have also been used in animal husbandry and aquaculture for growth promotion, feed efficiency improvement, prophylaxis as well as in the treatment of infectious diseases. From the animal welfare perspective, the use of antimicrobials improves the general health of farm animals and the hygiene of farming environments [1]. The agricultural food industry benefits from the use of antimicrobials for food-animal production and crop protection. In United States, nearly 80% of antibiotics produced are used in animal husbandry [2]. It was estimated, that globally each kilogram of meat harvested from cattle, chickens and pigs would lead to the consumption of 45 mg, 148 mg, and 172 mg of antimicrobials respectively, which is expected to increase by 67% from 2010 to 2030 [3].

Anti-microbial resistance is a recognized public health concern since its emergence limits the therapeutic options available to both clinicians and veterinarians. The first economic report on the impact of AMR proposed that if nothing was done, AMR-related deaths would increase from 700,000 to 10 million annually by 2050. It would cost trillions of USD in healthcare industry [4]. The improper use of antimicrobials for purposes other than treatment of infections has resulted in the selection for AMR in food production environments. Bacteria develop de novo resistance due to exposure to sub-inhibitory levels of antibiotics in their surroundings or directly acquire resistance mechanisms from other bacteria via, Horizontal Gene Transfer (HGT) [5].

Although widespread AMR has been mostly attributed to the selective pressure generated by overuse and misuse of antimicrobials, concerns have been raised based on recent growing evidences regarding co-selection for AMR among bacteria exposed to non-antibiotic compounds used in agri-food industry, such as biocides used as disinfectants, antiseptics and preservatives, heavy metals existing in nature and used in agricultural production [6]. The use of antimicrobials, heavy metals and biocides in food and agriculture has direct as well as indirect effects on the development of AMR in bacteria which can enter the food chain. Increasing unrest among public about antimicrobials usage in farming practices and the emergence of Multi-drug Resistant Bacteria has placed pressure on the agricultural food industry to act. A major area under scrutiny is the livestock food chain, from farms through slaughter houses and processing plants food to packaging and retail facilities [7]. This review will summarize the major factors in the selection and dissemination of food borne AMR along the food chain.

## Selection of AMR by using antimicrobials

### Mechanisms of AMR and pre-existence of antimicrobial resistance genes (ARGs)

Antimicrobial resistance includes two levels of resistance, the cellular level resistance and blocking of antimicrobial target and reduce entry of antimicrobials into or active efflux of antimicrobials out of the bacterial cell [2]. Reduced susceptibility of an organism to an antimicrobial may be innate (due to features of the microbe’s cell envelope, energy metabolism or the presence of an alternative metabolic pathway). It is also acquired via single or multi-step mutation that affects the target site and the effective concentration of the antimicrobial within the cell, or by the acquisition of genetic element encoding a feature such as an inactivating enzyme or an alternative to the target molecule i.e. HGT of resistance determinants. Table 1 show the representative mobile ARGs which are transferable between different bacterial strains and species. The community level resistance (biofilms and persisters) is also an issue causing antimicrobial therapy difficulties [26].

**Table 1:** Mobile antimicrobial resistance genes

Antibiotic Class	Mechanisms of resistance	Gene	Gene location	Species	Reference
β-lactams	Drug degradation: β-lactamases	Class A: Serine Penicillinases: TEM, SHV, CTX-M; Carbapenemases: KPC, IMI-2, GES	Plasmid	Multiples species of *Enterobacteriaceae*, *Acinetobacter* and *Pseudomonas*	[8–10]
		Class B (Metallo-β-Lactamases): NDM-1, IMP, VIM, NDM-9		*Stenotrophomonas maltophilia* and *Enterobacteriaceae* (NDM-1), *Klebsiella variicola* (NDM-9)	
		Class C (Cephalosporinases): AmpC		*Enterobacteriaceae* and *Pseudomonas*	
		Class D (oxacillinases): OXA-23, OXA-48, OXA-181, OXA-143, OXA-372		*Acinetobacter*, *Enterobacteriaceae, Aeromonas, Citrobacter freundii*	
		Class A: GES-1,VEB-1	Integron	*K. pneumonia*, *P. aeruginosa* and *A. baumannii*	
		Class B: NDM-1, IMP, VIM,		*Stenotrophomonas maltophilia*, *Enterobacteriaceae* and *A. baumannii* (NDM-1)	
Aminoglycosides	Drug modification	Nucleotidyltransferases: ANT(6)-Ia, ANT(9)-Ib, ANT(4′)-Ia C, ANT(4′)-IIa ANT(6)-Ib, ANT(4′)-IIb, ANT(9)-Ia, ANT(2″)-Ia, ANT(3″)-Ia *aadA31*	Plasmid Transposon Plasmid, transposon, integrin Integron	*Staphylococcus epidermidis, S. aureus, E. faecium, Streptococcus suis, P. aeruginosa, A. baumannii, P. aeruginosa, Vibrio cholera, Salmonella spp. S. enterica, E. coli, Aeromonas media, Pasteurella multocida, Yersinia enterocolitica, C. glutamicum, B. subtilis* *Pasteurella multocida* and *Histophilus somni*	[11, 12]
		Phosphotransferases: APH(4)-Ia, APH(6)-Id, APH(3′)-Ib, −IIIa C, −Via, −VIb, −VIIa, APH(2″)-Ia, −IIIa C APH(6)-Ic, APH(3′)-Ia, −IIa C APH(3′)-Ic, APH(2″)-Ie, APH(3″)-Ib	Plasmid Transposon Plasmid, transposon	*E. coli, S. enterica, P. aeruginosa, K. pneumoniae, Salmonella spp., Pseudomonas spp., V. cholerae, Edwardsiella tarda, Pasteurella multocida, Aeromonas bestiarum, A. baumannii, S.marcescens, Corynebacteriumspp., Photobacterium spp., Citrobacter spp. S. aureus, Enterococcus spp. E. casseliflavus*	
		Acetyltransfera**se**s: AAC(3)-Ia C, −Ib, −Ic, −Id, −Ie, −Ib, AAC(6′)-Ib” AAC(3)-IIa, −IIb, −IIc, −IVa, VIa AAC(6′)-Ia, −Ib C, −Ib’, −Ie, −If, −Ih, −Ip, −Iq, −Im, −Il, −Isa, −Iad, −Iae, −Iaf, −Iai, −Ib, − 31, − 32, − 33, −I30, −IIa, −IIb, −IIc, −Ib-cr, −Ie-APH(2″)-Ia, − 30/AAC(6′)-Ib’, ANT(3″)-Ii-AAC(6′)-IId	Integron Plasmid Plasmid, transposon, integron	*P. aeruginosa, P. fluorescens, S. enterica, E. coli, E. cloacae, Salmonella typhimurium, Proteus mirabilis, E. faecalis, E. faecium, Streptomyces albulus, C. freundii, A. baumannii, S. marcescens, Actinobacillus pleuropneumoniae, S. typhimurium, Citrobacter freundii*	
	Target modification: 16S rRNA methyltransferase	*armA, rmtB, rmtC, rmtH*	Plasmid	*K. pneumonia, E. coli, S. enterica, P. stuartii,* *E. aerogenes,*	[13]
		*armA, rmtA, rmtB, rmtC, npmA, rmtD, rmtE, rmtD2*	Transposon/integron	*C. freundii, P. aerugonosa, S. marcescens, P. mirabilis, E. coli,*	
Quinolones	Drug modification	Acetyltransferase: *aac(6′)-Ib-cr*	Plasmid	Multiple species of *Enterobacteriaceae*	[14]
	Target protection	*qnrA, qnrB, qnrS, qnrC, qnrD, qnrVC*	Plasmid	Multiple species of *Enterobacteriaceae*, also *Acinetobacter*, *Aeromonas*, *Pseudomonas*, and *Vibrio spp*.	
	Efflux pumps	*oqxAB, qepA*	Plasmid	Multiples species of *Enterobacteriaceae*	
Macrolides	Efflux pumps	*mefB* *mefC*	Plasmid	*E. coli* marine bacteria including *Vibrio* and *Photobacterium*	[15, 16]
		*mefI*	Transposon	*S. pneumoniae*	
		*mefA, mefE*	Integron /transposon	*Streptococcus, Staphylococci*	
		msr(A)	Plasmid	*Staphylococci*	
	Drug modification	Phosphotransferase: *mphC, mphA, mphE*	Plasmid	*S. aureus, E. coli, Serratia marscescens*, *K. pneumonia*, *A. baumannii*, *E. coli*, *Citrobacter freundii*	
		Esterase: *ereA, ereB*	Plasmid	*E. coli*	
	Target modification: 23S rRNA methylase	*erm*	Plasmid/transposon/integron	Multiple species	
	Ribosomal protection: ABC-F proteins	*msr(A)*	Plasmid	*staphylococci, enterococci, streptococci*	
Tetracyclines	Drug modification	*tetX,* *tet34* *tet37*		*Bacteroides, Aeromonas, Pseudomonas, Serratia, Vibrio*	[17]
	Ribosomal protection	*tetM, tetS, tetT, tetB(P), tetQ, tetW, tet32, tet36, otrA*	Transposon	*Acinetobacter, Afipia, Enterobacter, Erysipelothrix, Escherichia, Klebsiella, Lactobacillus, Lactococcus, Microbacterium, Mitsuokella, Mycobacterium, Neisseria, Prevotella, Porphyromonas, Ralstonia, Photobacterium, Pseudomonas, Selenomonas, Streptomyces, Vibrio, Megasphaera, Neisseria, Lactococcus, Lactobacillus, Veillonella, Actinomyces, Arcanobacterium, Bacillus, Butyrivibrio, Clostridium, Megasphaera, Roseburia, Staphylococcus, Bacteroides*	
		*tetO, poxtA*	Plasmid/transponson		
	Efflux pump	*tetA, tetB tetC, tetD, tetE, tetG tetH, tetJ, tetK, tetL tetA(P), tetV tetY, tetZ, tet30, tet31, tet33, tet35, tet38, tet39* *tcr3* *otrB, otrC*	Plasmid/ Transposon	*Acinetobacter, Haemophilus, Veillonella, Acinetobacter, Brevundimonsa, Neisseria, Photobacterium, Pseudomonas, Aeromonas, Chlamydia, Alteromonas, Escherichia Providencia, Actinobacillus, Moraxella, Pasteurella, Lactobacillus, Norcardia, Streptomyces, Morganella, Norcardia, Salmonella, Veillonella, Corynebacterium, Stenotrophomonas, Vibrio, Staphylococcu*	
	Unknown	*tetU*		Staphylococci	
Lincosamides	Drug modification: nucleotidyltransferases	*lnuA* *lnuB* *lnuC* *lnuE* *lnuF, linG* *linA*_*N2*_ *lnuG*	Plasmid Plasmid/integron Transposon Transposon	Staphylococci Staphylococci, streptococci, *Erysipelothrix rhusiopathiae* *S. agalactiae, Haemophilus parasuis* *Streptococcus suis, S. aureus* *E. coli, Salmonella enterica* *Bacteroides* *Enterococcus faecalis*	[18–20]
	Target modification: 23S rRNA methylase	*cfr*	Plasmid	Staphylococci, *Bacillus spp., Enterococcus spp., Macrococcus caseolyticus, Jeotgalicoccus pinnipedialis, E. coli*	
		*erm*	Plasmid/transposon/integron	Multiple species	
	Ribosomal protection: ABC-F proteins	*vga* *lsa*	Plasmid/transposon	Staphylococci, enterococci, streptococci	
	Efflux pump	*lsa(B)*	Plasmid	Staphylococci	
		*lsa(E)*	Integron		
		*vga(A), vga(E)*	Transposon/ plasmid	Staphylococci, streptococci, enterococci	
		*vga(C)*	Plasmid		
		*sal(A)*	Integron	Staphylococci	
Phenicols	Drug modification: Acetyltransferase	*catA* *catB*	Plasmid/ transposon Integron/ transposon	Multiple species of Gram-positive and Gram-negative bacteria Multiple species of Gram-negative bacteria	
	Target modification: 23S rRNA methylase	*cfr*	Plasmid	Staphylococci, *Bacillus spp., Enterococcus spp., Macrococcus caseolyticus, Jeotgalicoccus pinnipedialis, E. coli*	[21]
	Efflux pump	*optrA*	Plasmid	Enterococci	[19, 22]
		*cmr, cmx*	Plasmid/ transposon	*Corynebacterium spp., Rhodococcus spp.*	
		*floR*	Plasmid/ integron	*E. coli, K. pneumoniae, Pasteurella multocida, Pasteurella trehalosi, A. pleuropneumoniae, Stenothrophomonas maltophilia, P. multocida*	
		*fexA*	Transposon	Staphylococci	
		*fexB*	Plasmid	Enterococci	
		oqxAB	Plasmid	Multiple species of *Enterobacteriaceae*	
Streptogramin	Drug modification	Streptogramin A acetyltransferase: *vat(A), vat(B), vat(C)* *vat(D), vat(E), vat(H)*	Plasmid	Staphylococci Enterococci	[19]
		Streptogramin B lactone hydrolase: *vgb(A), vgb(B)*	Plasmid	Staphylococci	
	Target modification: 23S rRNA methylase	streptogramin A: *cfr*	Plasmid	Staphylococci, *Bacillus spp., Enterococcus spp., Macrococcus caseolyticus, Jeotgalicoccus pinnipedialis, E. coli*	
		streptogramin B: *erm*	Plasmid/transposon/intergron	Multiple species	
	Ribosomal protection: ABC-F proteins	streptogramin B: *msr(A)*	Plasmid	Staphylococci, enterococci, streptococci	
	Efflux pump	streptogramin A: *vga, lsa(A), sal(A)*	Plasmid/transposon/intergron	Staphylococci, enterococci, streptococci	
		streptogramin B: *msr(A), msr(C)*	Plasmid/integron		
Polymyxin	LPS modification	Phosphoethanolamine transferase: *mcr-1, −2, −3, −4, −5, −6, −7,* and *− 8*	Plasmid	*E. coli*, *K. pneumonia*, *Salmonella*, *Shigella sonnei*, *Enterobacter, Cronobacter sakazakii*, *Kluyvera ascorbata*	[23, 24]
Vancomycin	Target modification	*vanA–G*	Plasmid/transposon/integron	Staphylococci, enterococci, streptococci, *Oerskovia turbata, Arcanobacterium haemolyticum*	[25]

Antimicrobial resistance however, did not originate as a product of agricultural antimicrobial use. Antibiotic resistance is an ancient bacterial trait, existing in soil bacteria (the soil resistome) and carried on plasmids such as serine β-lactamases, millions of years before the dawn of agriculture [27]. Recent work has uncovered resistance in ancient permafrost, isolated caves, and in human specimens preserved for hundreds of years [28]. It had been shown that gene-encoding resistance to β-lactam, Tetracycline, and Glycopeptide antibiotics was present in metagenome samples of 30,000-year-old permafrost [29]. The gut microbiome of a pre-Columbian Andean mummy (dating of 980–1170 AD) was recently found to harbor β-lactam, Fosfomycin, Chloramphenicol, Aminoglycoside, Macrolide, Sulfa, Quinolones, Tetracycline, and Vancomycin resistance genes [30]. In a screen of sample of the culture-able microbiome of Lechuguilla Cave isolated for over 4 million years, the surface microbes were highly resistant to antimicrobials and some strains were resistant to 14 different commercially available antimicrobials including daptomycin and macrolide [31]. The results of these studies gave direct experimental evidence that AMR is ancient, and provided a glimpse into the evolutionary history of a natural environmental phenomenon.

### Selection of AMR in mutant selection window and sub-inhibitory concentrations

The concentration of an antimicrobial, either in the Mutant Selection Window (MSW) or below the minimum inhibitory concentration (MIC) of a wild-type population (also called sub-inhibitory concentration or sub-MIC concentration) is important for the selection of AMR [32]. MSW is a concentration range between the lowest concentration that exerts selective pressure, often approximated by the minimal concentration that inhibits colony formation by 99% (MIC99) and the MIC of the least drug-resistant mutant subpopulation, a value called the mutant prevention concentration (MPC) [33]. Drug-resistant mutant subpopulations present prior to the initiation of antimicrobial treatment are enriched and amplified when antimicrobial concentrations fall within the MSW.

Antimicrobials at sub-inhibitory concentrations (concentrations below MIC) are found in many natural environments like soil and water. Sub inhibitory concentrations are also generated as a result of antimicrobial therapy in humans and livestock (suboptimal dosing therapy, poor pharmacokinetics, usage of low-quality drugs, and a poor patient compliance) as well as administered as a feed additives to promote growth of animals [5]. In sub-MIC concentrations, the susceptible strains continue growing at a reduced growth rate, and the lowest antimicrobial concentration needed to choose for the resistant mutant over the wild type is called The Minimal Selective Concentration (MSC), from which to MIC the selection for the resistant mutants occurs [34]. Beside the pre-existed resistant mutants, de novo bacterial resistance may be promoted through sub-therapeutic antimicrobial concentrations by inducing non-specific mutagenesis resulting from stimulating the production of Reactive Oxygen Species [35].

### Selection of ARGs in food production system

Antimicrobial feeding in food animals has been as a selective force in the evolution of their intestinal bacteria, particularly by increasing the prevalence and diversity of ARGs [27]. However, the association between antimicrobial use and selection of resistance determinants is not as direct as often presumed. A recent study done in Danish pig farms demonstrated that the effect of antimicrobial exposure on the levels of seven ARGs (*ermB*, *ermF*, *sulI*, *sulII*, *tetM*, *tetO*, and *tetW*) was complex and unique for each individual gene. Several antimicrobial classes had both negative and positive correlations with the ARGs, indicating that antimicrobial exposure is not the only important determinant of the ARG levels [36]. In American swine production system, Ceftiofur is often administered to piglets at birth with males receiving a second dose at castration, and this operation may provide the selection pressure required for the dissemination of Carbapenemase-producing *Enterobacteriaceae* [37]. In *Campylobacter jejuni* isolates from beef cattle in confined feeding operations in Southern Alberta Canada, selection for resistance to fluoroquinolones was subtype dependent, whereas selection for resistance to tetracycline’s was not [38]. It was shown that the development of ciprofloxacin resistance was quite different among different serovar strains, due to the different mutation frequency and ciprofloxacin accumulation level [39].

## Co-selection of AMR by using non-antimicrobial compounds

Widespread AMR is mostly attributed to the selective pressure by overuse and misuse of antimicrobials. However, concerns have been raised based on growing evidences regarding co-selection of AMR among bacteria exposed to biocides which are used as disinfectants, antiseptics, preservatives and various cationic heavy metals included in animal diets as nutritional supplements, growth promoters and therapeutic agents for livestock [6]. These metals can also be spread on pastures to support crop growth and protection.

### Co-selection of AMR by heavy metals

Heavy metals occur everywhere in the environment, and on occasion at high concentrations in certain settings when they are used in agriculture production for various purposes. Heavy metals can continue to exist in the environment and remain stable for prolonged periods. While most veterinary antimicrobial compounds can be metabolized and cleared from the food-producing animals within weeks or months. The bioavailability of commonly feed-used minerals (mostly inorganic) is usually quite low in animals, and the unabsorbed heavy metals are excreted as fecal material in higher concentrations than in feeds [40].

The correlation between heavy metal tolerance and AMR had already been observed several decades ago. Copper (Cu) has been reported to be related to resistance against Ampicillin, Sulphanilamide [41], Erythromycin [42], Enrofloxacin [43], Vancomycin [44], and Glycopeptide [45]. Methicillin-resistant *Staphylococcus aureus* (MRSA) is often associated with Zinc (Zn) [45–48] and Cu [45]. There are positive correlations between Mercury (Hg) tolerant gene *merA* and transposon Tn21 [42]. *sulA* and *sulIII* were strongly correlated with levels of Cu, Zn and Hg [49]. Multidrug-resistant CTX-M-(15, 9, 2) and KPC-2-producing *Enterobacter hormaechei* and *E. asburiae* are found to possess a set of acquired Silver (Ag) resistance genes [50]. Other heavy metals including Nickel (Ni), Cadmium (Cd), and Chromium (Cr) are also reported to co-select certain AMR [42, 51–53]. A recent study showed that genes potentially conferring metal-resistance, including *arsA* (Arsenic compounds), *cadD* (Cd), *copB* (Cu) and *czrC* (Zn/Cd) were frequently present in livestock associated MRSA [54]. A Chinese study even found only a weak positive correlation between ARGs and their corresponding antimicrobials, while significant positive correlations were found between some ARGs (*sulA* and *sulIII*) and typical heavy metals such as Hg, Cu, and Zn [49].

The molecular mechanisms for the ability of bacteria to develop heavy metal resistance are similar to those for AMR since heavy metals have known antimicrobial effects [55]. Co-selection is achieved in two ways: (1) Co-resistance, whereby selection for one gene fosters the maintenance of another resistance gene and (2) Cross-resistance, whereby one resistance gene can offer protection from multiple toxic chemicals [56]. Co-resistance/Co-transfer for a heavy metal and an antimicrobial is often caused by the co-resident metal and antimicrobial- resistance genes, which can be physically localized to plasmids or chromosomes that also contain one or more ARGs [57, 58]. For example, MRSA from livestock have been described harboring plasmids carrying resistance genes for Cu and Cd (*copA*, *cadDX* and *mco*) and for multiple antimicrobials including Macrolides, Lincosamides, Streptogramin B, Tetracyclines, Aminoglycosides and Trimethoprim (*erm(T)*, *tet(L)*, *aadD* and *dfrK*) [59]. The link between Zn usage in animal feeds and the occurrence of MRSA is explained by the physical presence of the Zn resistance gene, *czrC*, on the methicillin resistance-encoding SCC*mec* element [60, 61]. Another example of co-resistance involved a number of resistance genes such as *aadA2* (streptomycin^R^), *qacED1* (spectinomycin^R^) and *sul1* (sulfonamide^R^) located to Tn5045 where chromate resistance genes *chrBACF* are found [62]. A Portuguese study found in monophasic *S. Typhimurium* variants of human and pig origin that ARGs in this multi-drug-resistant Pathovar were co-located with *sil* operon which encoded an efflux for Cu and Ag on the chromosome or a non-transferable plasmid [63]. A conjugation assay demonstrated co-transfer of *tcrB* and *erm(B)* genes between *E. faecium* and *E. faecalis* strains [64]. Genomic analysis of *E. faecalis* from Cu-supplemented Danish pigs revealed the presence of chromosomal Cu-insusceptibility genes, including the *tcrYAZB* operon and Tetracycline (*tetM*) and Vancomycin (*vanA*) resistance genes were present in one of the “Cu-insusceptible” isolates [65]. The genetic linkage of Cu, Zn and ARGs in bacteria has been comprehensively summarized in a recent review written by Keith Poole [57].

Like antimicrobials, metals are stressors that activate a variety of adaptive/protective responses in bacteria, and this can make co-regulation of metal and antimicrobial resistance resulting in cross-resistance [66]. In Gram-negative bacteria, The Membrane Stress Responsive Two Component System CpxRA which is linked to resistance against variety of cell envelope-targeting drugs [67] is also Cu-responsive and contributes to Cu tolerance [68]. In the presence of Zn, TCS CscRS in *Pseudomonas aeruginosa* influences the transcription of *czcCBA* operon encoding an RND-type efflux pump which confers resistance to Zn, Cd and cobalt (Co), meanwhile the CscRS system also reduces the expression of porin OprD through which imipenem enters the bacteria [69]. In *Listeria monocytogenes*, a Multidrug efflux pump MdrL confers resistance against a range of antimicrobials, and the same transport system also works for heavy metals such as Zn, Co and Cr [70]. Similarly, the Envelope Stress Response Sigma Factor RpoE activated by Polymyxin B and linked to Polymyxin B resistance in a number of Gram-negative bacteria [71] is also activated by Zn in *E. coli* and contributes to Zn and Cu tolerance [72]. Cu has also been shown to increase expression of the Oxidative Stress-responsive Regulatory Gene *soxS* that is linked to expression of the AcrAB efflux pump and multidrug resistance in *E. coli* [73].

Biofilms, in which bacteria are embedded in extra cellular polymeric substances, are more resistant to heavy metals than their planktonic counterparts [74]. In turn, the biofilm matrix may drive the frequency of mutation in the bacterial genomes, which is favorable for co-selection for AMR [75]. Many reports have described in several Gram-negative bacteria that Cu induces a Viable but Nonculturable (VNC) state, which is a stress-induced antimicrobial-resistant dormant state [76]. A Zn-linked VNC state has also been seen in *Xylella Fastidiosa*, and it appears to hasten the onset of the VNC state in this organism [77]. Moreover, the exposure of *E. coli* to Cu has been shown to increase the recovery of small colony variants, and the slow-growing variants are typically antimicrobial-resistant for a variety of bacteria [78].

Heavy metals can also facilitate the HGT. A recent study suggested that sub-inhibitory concentrations of heavy metals accelerate the horizontal transfer of plasmid-mediated ARGs in water environment by promoting conjugative transfer of genes between *E. coli* strains [79]. Another study showed that via Cu shock at 10 and 100 mg/L loading on bacteria from a drinking water bio-filter, bacterial resistance to Rifampin, Erythromycin, Kanamycin, and a few others was significantly increased. Furthermore, the relative abundance of most ARGs, particularly the mobile genetic elements (MGE) *intI* and transposons, were markedly enriched by at least one-fold [80].

### Co-selection of AMR by biocides

Biocides can be used as antiseptics on body surfaces, as disinfectants on equipment and surfaces in many environments including farms and hospitals, as decontaminants on carcass surfaces following slaughter, and as preservatives in pharmaceuticals, cosmetics and food [81]. A possible cross-resistance between biocides and antimicrobials is still controversial. Some studies have reported that there is no cross-resistance between biocides and antimicrobials. For example, no cross-resistance between Chlorhexidine and five antimicrobials was found in 130 *Salmonella* spp. from two Turkey farms [82]. Among 101 genetically distinct isolates of *Burkholderia cepacia*, no correlation was found between the susceptibility to Chlorhexidine and 10 different antimicrobials [83]. On *Enterococcus faecium*, low doses of Peracetic Acid, usually used as disinfectant in wastewater treatments, promoted a bacterial adaptation but without affecting the abundance of the AGRs [84].

On the other hand, several surveys have been performed on the co-selection of AMR by biocides in bacterial isolates from food-animals and aquacultures. It has been indicated that the overall exposure to Chlorhexidine Digluconate increases the risk for resistance to a variety of antimicrobials [85]. When 310 Gram-positive isolates from milking cow teats were subjected to Iodine or Chlorhexidine antisepsis, a significant association among *Streptococci* between reduced susceptibility to Chlorhexidine and to Ampicillin, Tetracycline and three Aminoglycoside antibiotics [86]. In 87 isolates from seafoods, moderate positive correlations were detected for the biocides Cetrimide, Hexadecylpyridinium chloride and Triclosan with the antibiotic Cefotaxime, and also for Triclosan with Chloramphenicol and Trimethoprim/Solfamethoxazole and with the phenolic compound Thymol [87]. It was reported in *E. coli* O157 and various *Salmonella* serovars reductions in susceptibility to a panel of antimicrobials following stepwise training of Triclosan, Chlorhexidine and Benzalkonium chloride [88]. Exposure of veterinary field *E. coli* isolates to three quaternary ammonium compounds yielded elevations of MIC that were above the clinical breakpoints for Phenicol, Tetracycline, Fluoroquinolone, β-lactams and Trimethoprim [89]. *Salmonella Enteritidis* surviving a short exposure to in-use concentrations of Chlorine exhibited up to eight-fold increases in MIC values for Tetracycline, Nalidixic Acid and Chloramphenicol [90], similar to those observed with stepwise training procedures.

There are more surveys and investigations that have involved hospitals or other healthcare environments about the co-selection of AMR by biocides [6]. When the aerobic microbial communities were exposed to Benzalkonium Chloride, the community-wide MIC values for Benzalkonium Chloride, Ciprofloxacin, Tetracycline and Penicillin G were all increased [91]. Recent data showed that exposure of vancomycin-resistant *E. faecium* to Chlorhexidine for only 15 min up-regulates the *vanA*-type Vancomycin resistance gene (*vanHAX*) and genes associated with reduced Daptomycin susceptibility (*liaXYZ*) [92].

It has been demonstrated a role of efflux for the co-selection of AMR in some biocide training studies [93], and reduced susceptibility to biocides may follow from the development of AMR vice versa [94–96]. Under Benzalkonium Chloride exposure, the expression of two non-specific efflux pumps genes (*lde* and *mdrL*) in *Listeria monocytogenes* isolated from pork meat processing plants was evaluated [97]. The expression of *lde* was dose-dependent in the case of the post cleaning and disinfection procedure strain, while the expression of *mdrL* was inhibited under low biocidal stress (10 ppm) and enhanced in the presence of high stress (100 ppm). In a study of biofilm formation potential and efflux pump activity, *E. coli* isolates from dairy equipment that had reduced susceptibility to Benzalkonium Chloride and Ciprofloxacin proved to have superior biofilm capacity, in parallel with increased efflux activity [98]. Improved biofilm capability plus efflux has also been seen in Triclosan-adapted *E. coli* [99]. Genetic co-occurrences suggest that plasmids provide limited opportunities for biocides and metals to promote horizontal transfer of AMR through co-selection, whereas quite large possibilities exist for indirect selection via chromosomal biocide/metal resistance genes [100].

There are a lot of theoretical and experimental evidences that certain biocides may co-select for AMR, mainly by close link of biocide resistance determinants to AMR determinants. However, there is lack of empirical data to indicate that the use of biocides drives this co-selection of AMR in the food chain [101, 102].

## Transfer and dissemination of AMR in food chain

The environmental resistome comprises both the natural AMR pool and contaminant AMR pool resulting from human activities [103]. The transfer of ARG from natural reservoirs to other bacteria may be a rare and random event, contaminant ARBs and ARGs may be able to spread rapidly and widely ((e.g. New Delhi metallo-beta-lactamase, blaNDM-1 [104]; extended-spectrum beta-lactamase blaCTXM-15 [105]; MRSA [106]).

### Transmission of AMR from food animals to the environments

Microbiomes encounter low-doses or sub-therapeutic levels of antimicrobial agents from all mechanistic classes in food animal production. This modern practice exerts broad effects on the gut microbiome of food animals, which is subsequently transferred to animal waste. Land application of animal manure is a common agricultural practice potentially leading to dispersal and propagation of ARGs in environmental settings. Many studies have proved that MGEs and ARGs are closely associated in their persistence in the composts under antimicrobial selection [107]. Different manure sources may influence the fate of resistome in agro-ecosystems as shown recently in a study demonstrating that application of swine and poultry manures might enrich more soil ARGs than cattle manure, and the relative abundance of ARGs had significantly positive correlations with integrase and transposase genes [108]. A study compared 864 metagenomes from humans, animals and external environments and found that water, sediments and soil generally carried low relative abundance and few varieties of known ARGs, furthermore the wastewater/sludge was on par with the human gut, indicating that the environments with the largest relative abundance and/or diversity of ARGs were those subjected to industrial antibiotic pollution [109].

In food animals, ARBs are usually developed in animals’ bodies (especially in the gastrointestinal tract) after using antibiotics. Differently, AMR in fruits, vegetables and other foods of plant origin is often due to the contamination with ARBs and ARGs along the food chain, from primary production to consumption [110]. Important sources of microbial contamination in the pre-harvest environment include soil, organic fertilizers and irrigation water.

Transduction is a significant mechanism of horizontal gene transfer in natural environments, which has traditionally been underestimated as compared to transformation. A study found that soil phages were the most versatile in terms of ARG carriage, and the phages from organized farms showed varied ARGs as compared to the unorganized sector [111]. Another study screened pig feces from three commercial farms for 32 clinically relevant ARG types and found that bacteriophage DNA contained 35.5% of the target ARG types and *sul1*, *bla*_*TEM*_ and *ermB* were found in 100% of the phage DNA samples [112]. Using the ratio index of the abundance of ARGs in bacteriophages and bacteria as an estimator of bacteriophage ability to transmit ARGs, it was found that the ratio for *qnrA* was the greatest (about 10^− 1^) and differed from the most abundant bacteriophage ARG *ermB*, and *fexA* not *floR* had the lowest ratio value (about 10^− 6^).

### Transmission of AMR from the environments to humans

The antimicrobial resistome is harbored by; (i) Antimicrobial-resistant bacteria called carriers that can spread ARG in the environment, but cannot colonize or infect the human or animal body.

(ii) Antimicrobial-resistant bacteria called vectors that can colonize and sometimes invade the human or animal body [103]. Even though carriers are not able to colonize and infect humans, their spread and proliferation in the environment would increase the abundance and diversity of ARG in vectors. Hence, it may increase the risks of transmission of ARB to humans. It should be noted that most vectors are not pathogens, because even if vectors can colonize the human body, they may lack crucial virulence genes and therefore unable to cause disease in a healthy host [103].

In searching a literature on the evidence for human exposure to extended-spectrum β-lactamase (ESBL) producing *Enterobacteriaceae,* MRSA, and Vancomycin-resistant *Enterococcus* spp. in the environment, a review paper published in 2015 found that ARBs were detected in the contamination sources (66/66) such as wastewater and manure, and no direct evidence was found for transmission to humans through the environment [113]. Although several studies performed on molecular typing of human and environmental isolates, only one obtained this level of evidence, but the direction of transmission could not be determined (environment transmitting AMR bacteria to humans or vice versa) [114].

### Transmission of AMR from animals to humans through food chain or close contacts

Many pathogens of animals are zoonotic, and therefore any development of resistance in pathogens associated with food animals may spread to humans through food chain. Human infections by antibiotic-resistant pathogens such as *Campylobacter* spp., *Salmonella* spp., *E. coli* and *S. aureus* are increasing [2].

The impact of animal reservoirs on human health remains debatable and unclear; nonetheless, there are some examples of direct links that have been identified. In ESBL/AmpC and Carbapenemase-producing *Enterobacteriaceae* occurring in animals, ESBL/AmpC- or Carbapenemase-encoding genes are most often located on MGEs favoring their dissemination [115]. In most African surveys, among ESBLs, certain *bla*CTX-M-15-harbouring clones (ST131/B2 or ST405/D) are mainly identified in humans. But these have also been reported in livestock species from Tanzania, Nigeria or Tunisia; international trade of poultry meat seems to have contributed to the spread of other ESBL variants, such as CTX-M-14, and clones [116]. Even though exposure to animals is regarded as a risk factor, evidence for a direct transfer of ESBL/AmpC-producing bacteria from animals to humans through close contacts is limited. The extent to which food contributes to potential transmission of ESBL/AmpC producers to humans is also not well established.

For heavy metals, associations have been identified between reduced Zn or Cu susceptibility and AMR among pig *Salmonella* isolates, which are foodborne pathogens [6]. Co-transmission of the Cu efflux-associated *tcrB* and erythromycin resistance *erm(B)* genes has been proved between a marine sediment-derived livestock species *Enterococcus hirae* and *E. faecalis* in conjugation experiments. The experiments highlighted the scope for AMR selection by the marine environment through heavy metals and its possible involvement in antibiotic-resistant enterococcal infections [117]. Moreover, there is reasonable evidence of a co-resistance phenomenon involving Cu, Macrolides and perhaps Vancomycin among Enterococci of pigs, whereas the relevance of this to disease-causing strains in humans remains undetermined [6].

## Conclusions and perspectives

The contribution of AMR originally selected for in the agricultural sector to resistance in human pathogens is not known exactly, but is unlikely to be negligible. Since dosing regimens are less controlled in agriculture than in human health care, veterinary and environmental microbes are often exposed to sub-inhibitory concentration of antimicrobials, which is considered as a risk factor for de novo resistance, transfer of ARGs, and selection for already existing resistance [118]. Based on the present knowledge, short treatments with the highest dose that does not cause unacceptable side-effects may be optimal for achieving therapeutic goals while minimizing development of resistance. Novel approaches such as combination or alternating therapy are promising, but need to be explored further before they can be implemented in daily practice.

Co-selection of genes that confer resistance to antimicrobials, biocides, heavy metals and other chemical hazards is a potentially ecologically and clinically important phenomenon. Non-antibiotic compounds used in agri-food production, such as antibacterial biocides and heavy metals, may also contribute to the promotion of AMR through co-selection. This may occur when resistance genes to both antimicrobials and metals/biocides are co-located together in the same cell (co-resistance), or a single resistance mechanism (an efflux pump) confers resistance to both antimicrobials and biocides/metals (cross-resistance), leading to co-selection of bacterial strains, or mobile genetic elements (MGEs) that they carry [119].

The agri-food industry is coming under pressure to reduce its usage of antimicrobial compounds. A recent study analyzing AMR and antibiotic consumption worldwide versus many potential contributing factors found that antibiotic consumption was not significantly associated with antimicrobial resistance index. This suggest that reduction of antibiotic consumption will not be sufficient to control AMR because the spread of resistant strains and resistance genes seems to be the dominant contributing factor [120]. Moreover, even when no antimicrobial compounds are used, certain heavy metals or biocides can maintain or even increase the bacterial resistance against certain agents [6]. Therefore, the effort of one nation to reduce its application of antimicrobial drugs in agri-food production alone will not yield the required outcome in terms of limiting consumer exposure. Resistant zoonotic agents and commensal strains carrying AMR genes reach the human population by a variety of routes, foodstuffs being only one of these [113]. Improving sanitation, increasing access to clean water, and ensuring good governance, as well as increasing public health-care expenditure and better regulating the private health sector are all necessary to reduce global antimicrobial resistance [120]. For agri-food industry, all countries must develop a code of practice to mitigate the risks to the consumer and preserve the existing valuable chemotherapeutic agents for future generations.

## Data Availability

Not applicable.

## Abbreviations

AMR: Antimicrobial resistance
ARB: Antimicrobial-resistant bacteria
ARG: Antimicrobial resistance gene
ESBL: Extended spectrum β-lactamase
HGT: Horizontal gene transfer
MGE: Mobile genetic element
MIC: Minimum inhibitory concentration
MRSA: Methicillin-resistant *Staphylococcus aureus*
VNC: Viable but Nonculturable
